# Access to malaria prevention and control interventions among seasonal migrant workers: A multi-region formative assessment in Ethiopia

**DOI:** 10.1371/journal.pone.0246251

**Published:** 2021-02-23

**Authors:** Mesele Damte Argaw, Asfawesen GebreYohannes Woldegiorgis, Habtamu Aderaw Workineh, Berhane Alemayhu Akelom, Mesfin Eshetu Abebe, Derebe Tadesse Abate, Eshetu Gezahegn Ashenafi

**Affiliations:** USAID Private Health Sector Project, Abt Associates Inc., Addis Ababa, Ethiopia; Instituto Rene Rachou, BRAZIL

## Abstract

**Background:**

Mobile or seasonal migrant workers are at increased risk for acquiring malaria infections and can be the primary source of malaria reintroduction into receptive areas. The aim of this formative assessment was to describe access to malaria prevention and control interventions among seasonal migrant or mobile workers in seven regional states of Ethiopia.

**Methods:**

A cross-sectional formative assessment was conducted using a qualitative and quantitative mixed-method design, between October 2015 and October 2016. Quantitative data were collected from organizations that employ seasonal migrant workers and were analyzed using Microsoft Excel and ArcGIS 10.8 (Geo-spatial data). Qualitative data were collected using in-depth interview from 23 key informants (7 seasonal migrant workers, and 16 experts and managers of development projects who had hired seasonal migrant workers), which were recorded, transcribed, translated, coded, and thematically analyzed.

**Results:**

There were 1,017,888 seasonal migrant workers employed in different developmental organizations including large-scale crop cultivating farms, sugar cane plantations, horticulture, road and house construction work, and gold mining and panning. Seasonal migrant workers’ housing facilities were poorly structured and overcrowded (30 people living per 64 square meter room) limiting the use of indoor residual spraying (IRS), and forcing seasonal migrant workers not to use long lasting insecticidal treated nets (LLINs). Seasonal migrant workers are engaged in nighttime activities when employment includes watering farmlands, harvesting sesame, and transporting sugar cane from the field to factories. Despite such high-risk living conditions, access and utilization of preventive and curative services by the seasonal workers were limited. Informal migrant worker employment systems by development organizations and inadequate technical and financial support coupled with poor supply chain management limited the planning and delivery of malaria prevention and treatment strategies targeting seasonal migrant workers.

**Conclusions:**

Seasonal migrant workers in seven regions of Ethiopia were at substantial risk of acquiring malaria. Existing malaria prevention, control and management interventions were inadequate. This will contribute to the resurgence of outbreaks of malaria in areas where transmission has been lowered. A coordinated action is needed among all stakeholders to identify the size of seasonal migrant workers and develop and implement a comprehensive strategy to address their healthcare needs.

## Introduction

Malaria is one of the deadliest infectious diseases worldwide [1]. In 2018 it accounted for an estimated 228 million cases and 405,000 deaths [2]. Africa is the most affected continent contributing to more than 90% of morbidities and mortalities resulting from malaria [2]. Across several sub-Saharan African countries, malaria is associated with travel-related risk factors posing a major challenge for control programs [3–7].

Ethiopia has made significant progress on malaria prevention and control over the past few decades. A scoping review that compared the periods between 2001–2010 and 2011–2016 reported a decline in the average number of annual malaria cases from 43.1 to 29.0 per 1000 population and deaths from 2.1 to 1.1 per 100 000 population [8, 9]. Despite these encouraging progresses, more than 66 million people in Ethiopia are still at risk of malaria infection. In 2019 alone, Ethiopia reported about 1 million cases and 213 deaths from malaria [1].

Seasonal migrant or mobile workers are disproportionally affected with malaria [3]. Ethiopia’s large development projects in agriculture, mining, construction, and energy are found in the malaria-endemic lowlands of the country. These projects attract and mobilize thousands of workers from the highlands. These mobile workforces are susceptible to malaria infection and transmission. Data from a five-year health record (from 2013–2017) in North West Ethiopia indicates that up to 40% of the reported malaria cases are only from three districts (Metema, West Armachiho and Quara) where there are an influx of seasonal farm workers [4]. The prevalence of malaria in young seasonal migrant workers in Northern Ethiopia is around 12% compared to 0.5% in the local population [1, 3]. Older boys and men are at special risk due to occupation and travel related exposure during seasonal farm work [3, 4, 6, 7, 10]. Several factors such as sleeping outdoors, lack of access to prevention and treatment interventions, and self-medication may contribute to this imbalance. In Ethiopia, only about 49% of migrant workers own long lasting insecticide treated bed nets [11, 12]. Self-medication is widely practiced among migrant or seasonal workers, largely contributing to the emergence and spread of malaria resistance to artemisinin and partner drugs [13].

What is more alarming is, that these mobile and seasonal workforces are further contributing to the resurgence of outbreaks of malaria when returning to their permanent residence areas where transmission has been lowered [6, 7]. Ethiopia’s malaria elimination strategy emphasizes the need to establish mobile test and treat stations and provide targeted mass drug administration (MDA) in places where seasonal migrant workers reside. However, there is no inter-sectoral collaboration policy that can support the implementation of this and other healthcare strategies relating to mobile and seasonal migrant workers in Ethiopia [14]. Reports indicate that a significant proportion of migrant workers tend to visit private healthcare facilities. A study in Northern Ethiopia revealed that 31.6% of seasonal migrant workers with a fever sought treatment at private hospitals/clinics [3].

The USAID Private Health Sector Project aims to improve standard malaria care services in the formal private health sector and in seasonal migrant and mobile populations. Here we report the result of a formative assessment conducted as a part of this project to evaluate seasonal migrant workers access to malaria prevention and control interventions. The data generated will provide empirical evidence for the development of malaria care service modalities and elimination strategies for seasonal migrant workers in Ethiopia.

## Materials and methods

### Study design

A cross-sectional formative assessment was conducted between October 2015 and October 2016 using qualitative and quantitative mixed-method design [15, 16].

### Study area

The assessment was conducted in seven (out of eleven) regional states of Ethiopia: Afar, Amhara, Benishangul-Gumuz, Gambella, Oromia, Southern Nations Nationalities and Peoples (SNNP), and Tigray. These regions were selected due to a) their higher malaria burden, and b) the larger presence of development projects employing seasonal migrant workers. Malaria incidences in these regions range from 104/1000 population in Oromia to 120/1000 population in the Gambella [1]

### Study participants and sampling

Purposive sampling techniques were employed. Study participants included: a) regional and district level malaria program experts from various sector offices (health, agriculture, labor, and social affairs, investment, etc.), b) managers or supervisors of developmental organizations and projects such as large-scale farms, gold mining, and construction sites, and c) migrant or seasonal workers. In this study, seasonal migrant and/or mobile workers were defined as employees who moved from their origin to destination districts for labor work for less than one year. People that did not consent to participate and were not available during the study period were excluded.

For the quantitative data, the seasonal migrant worker employing organizations in the seven regional states were targeted. Two thousand nine hundred and thirteen documentations from 93 organizations and government bureaus were collected.

For the qualitative data, a two-stage sampling process was employed. First, a list of workplaces where seasonal migrant workers reside was created in consultation with regional states sector bureaus from which districts and workplaces were selected. Second, the study participants were purposively selected for in-depth interviews. Qualitative data were collected from 23 key informants until the point of saturation, which was judged by the redundancy of information [15, 16].

### Data collection

Quantitative data were collected through interviewer-administered questionnaires and data abstraction forms. The data collection tools were developed after reviewing the relevant literature [3, 5]. These were then piloted in non-study districts. A three-step data collection process was employed. First, an inventory tool was used to collect data on the number and location of organizations or companies that employ seasonal migrant workers (S1 File). The inventory was administered to the departments of agriculture, investment, labor and social affairs, and health within the study regions. Second, a semi-structured questionnaire was administered to the managers and supervisors of the seasonal migrant workers to capture data on the type of business the organization is engaged in, size of land, and size, duration of employment, mobility patterns, housing conditions, and access to malaria prevention, control, and elimination services of seasonal migrant workers (S2 File). Third, migrant seasonal workers employment-related documents were reviewed. Geo-spatial data (district shapefiles) were obtained from the Ethiopian Central Statistical Agency (2016) (https://africaopendata.org/dataset/ethiopia-shapefiles) to map the number and destination districts of seasonal migrant workers.

Qualitative data were collected through in-depth interviews with key informants. In-depth interview guides were developed and piloted. This captured the lived experiences and opinions of seasonal migrant workers on factors that may enable or deter the implementation of malaria prevention and control interventions (S3 File). In addition, in-depth interviews were conducted with experts of the regional state health bureaus, zone health departments, districts health offices and to assess the burden of malaria in the region, and how the regions and districts are handling malaria prevention and control for seasonal migrant workers (S4 File).

### Data analysis

Quantitative data were checked for completeness, cleaned, and entered into Excel Database (Microsoft Office 2010, Microsoft Corporation, Redmond, Washington, USA 2010) [17], and a descriptive analysis was conducted (S5 File). Geo-spatial analysis was done using ArcGIS 10.8 by joining the number of seasonal migrant workers with the destination district shapefiles (S6 File) obtained from the Ethiopian Central Statistical Agency (2016) (https://africaopendata.org/dataset/ethiopia-shapefiles) [18].

The qualitative data were transcribed and translated into English. Transcripts were coded manually and collated into sub-themes. Then, a thematic analysis was conducted based on a conceptual framework of information usage in the coordination of care [19]. To ensure qualitative data quality, the data were interpreted by triangulation with information obtained from quantitative data and observations.

### Measures to ensure trustworthiness

The trustworthiness, of the study findings was ensured in the following ways. The study was led by persons with adequate expertise and firsthand experience in malaria prevention, control, and elimination. Quantitative and qualitative data were collected by trained data collectors using a rigorously piloted tool. Data were collected from a diverse group of study participants: seasonal migrant workers, managers, health team leaders of the seasonal migrant worker employing organizations, and experts of regional health bureaus of all seven malaria endemic regions of Ethiopia to enhance the generalizability of the findings. The data collectors spent adequate time with seasonal migrant workers and their employers to observe and understand context.

### Ethical considerations

Ethical clearance was obtained from Amhara, Oromia, Tigray, SNNP, and Benshangul-Gumuz regional state health bureau Institutional Review Boards (IRBs)–Ref. numbers: HRTT/1/337/08, BEFO/PHEM/1-8/2215, 2558/365/08, PH48-240/953, 275/M1/0009, respectively. Permission to collect data was obtained from sector offices and seasonal migrant workers employing organizations. Informed consent to conduct interviewer-administered data collection and audio recorded in-depth interviews were taken from all interviewees. To maintain the confidentiality of the collected data, anonymity was maintained throughout the research process.

## Results

### Quantitative component

#### Size of seasonal migrant worker population

**Fig 1:**
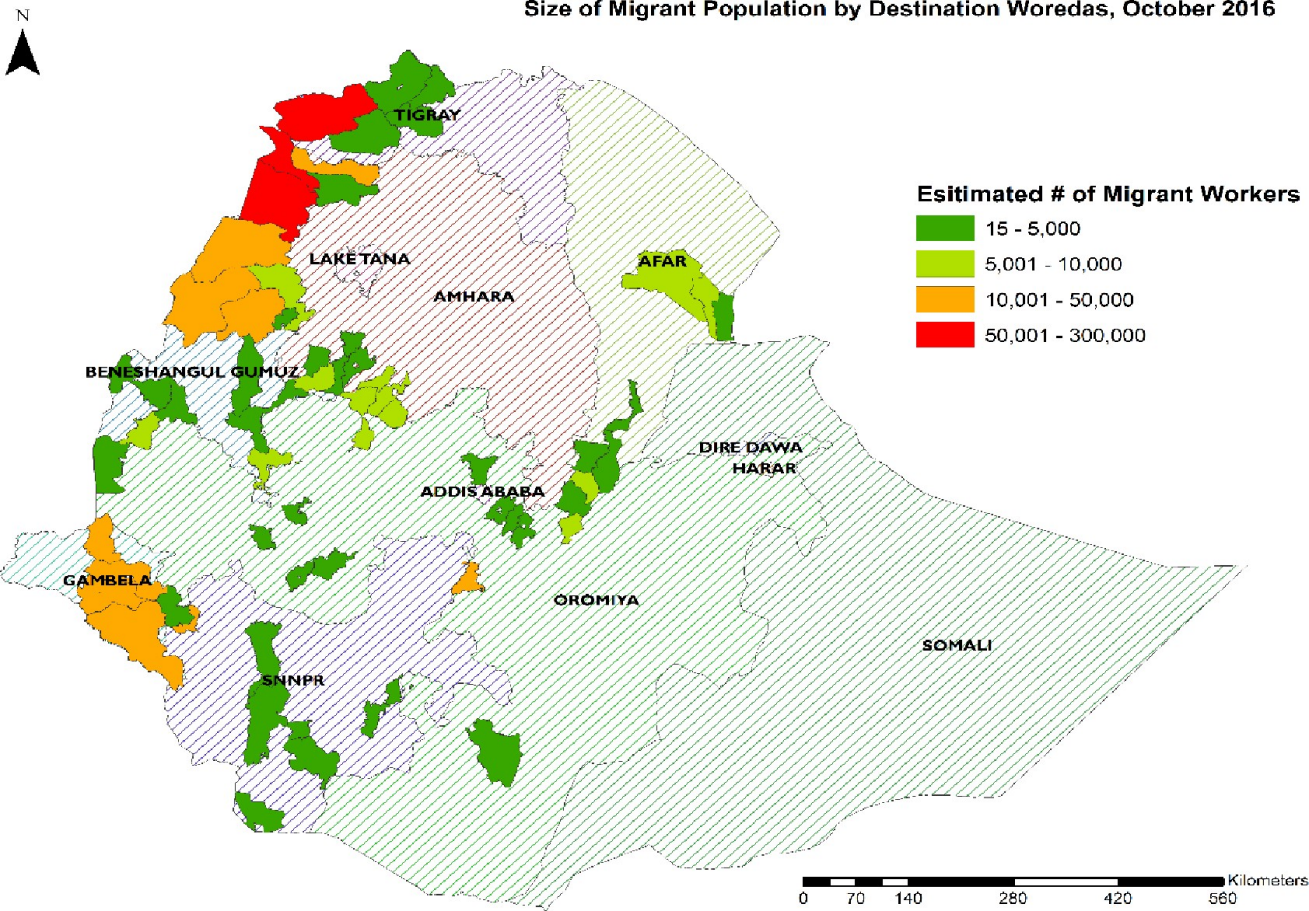
Number of seasonal migrant workers by destination districts, October 2016. Fig 1 presents a map of Ethiopia, with sizes of seasonal migrant workers in destination districts in four categories.

From the inventory data there were 1,017,888 seasonal migrant workers in seven regional states of Ethiopia. Of these, 102,314 (10%) were employed in 58 organizations selected for this study: 5 largescale construction sites; 9 floriculture and horticulture farms (ranging between 5,000 and 10,000 hectares), 13 government-owned large-scale sugar plantation farms (ranging between 4,000 and 25,000 hectares), and 14 private owned large-scale farms sesame farm, coffee plantations, groundnut, and biofuel farms (ranging between 5,000 and 50,000 hectares). About 43% (433,441) and 27% (271,299) of the seasonal migrant workers were employed in 10 districts of the Amhara and three districts of the Tigray regional states respectively (Fig 1). Seasonal migrant workers’ stay on their jobs ranged from 6–9 months.

The largest number of seasonal migrant workers (1,017,888), were employed between October and December, followed by between July and September (1, 009,697). The lowest employment period was from January to March and April to June with 99,030, and 96,399 migrant workers employed in the respective periods. Male seasonal migrant workers consisted of 91.2% of the workforce, while females accounted for 8.8%. Unlike the other six regions, the Oromia region had slightly higher numbers of female migrant workers (23.4%), (Table 1).

**Table 1:** Summary of seasonal migrant workers’ employment opportunities by business domain, region and sex, October 2016 (N = 1,017,888).

Region	Male	Female
Employing organizations	n (%)	n (%)
**Afar**	**25,565 (92.3)**	**2,124 (7.7)**
Floriculture and horticulture	10,089 (92.2)	854 (7.8)
Sugar cane plantation and factory	15,476 (92.4)	1,270 (7.6)
**Amhara**	**402,958 (93.0)**	**30,483 (7.0)**
Crop production (sesame, groundnut, sorghum, cotton etc.)	400,158 (93.2)	28,983 (6.8)
Sugar cane plantation and factory	2,800 (65.1)	1,500 (34.9)
**Benishangul Gumuz**	**57,183 (87.5)**	**8,197 (12.5)**
Crop production	57,183 (87.5)	8,197 (12.5)
**Gambella**	**122,739 (93.4)**	**8,736 (6.6)**
Crop production	122,739 (93.4)	8,736 (6.6)
**Oromia**	**59,773 (76.6)**	**18,232 (23.4)**
Construction	104 (99.0)	1 (1.0)
Crop production	42,049 (90.7)	4,314 (9.3)
Floriculture and horticulture	3,067 (25.1)	9,133 (74.9)
Gold mining	20 (100.0)	0 (0.0)
Sugar cane plantation and factory	14,533 (75.2)	4,784 (24.8)
**SNNP**	**8,602 (81.2)**	**1,997 (18.8)**
Construction	2,064 (92.3)	160 (7.2)
Crop production	3,763 (81.2)	872 (18.8)
Floriculture and horticulture	386 (66.1)	198 (33.9)
Sugar cane plantation and factory	2,389 (75.7)	767 (24.3)
**Tigray**	**251,046 (92.5)**	**20,253 (7.5)**
Crop production	247,886 (92.6)	19,855 (7.4)
Sugar cane plantation and factory	3,160 (88.8)	398 (11.2)
**Grand total**	**927,866 (91.2)**	**90,022 (8.8)**

#### Access to malaria diagnosis and treatment services

**Fig 2:**
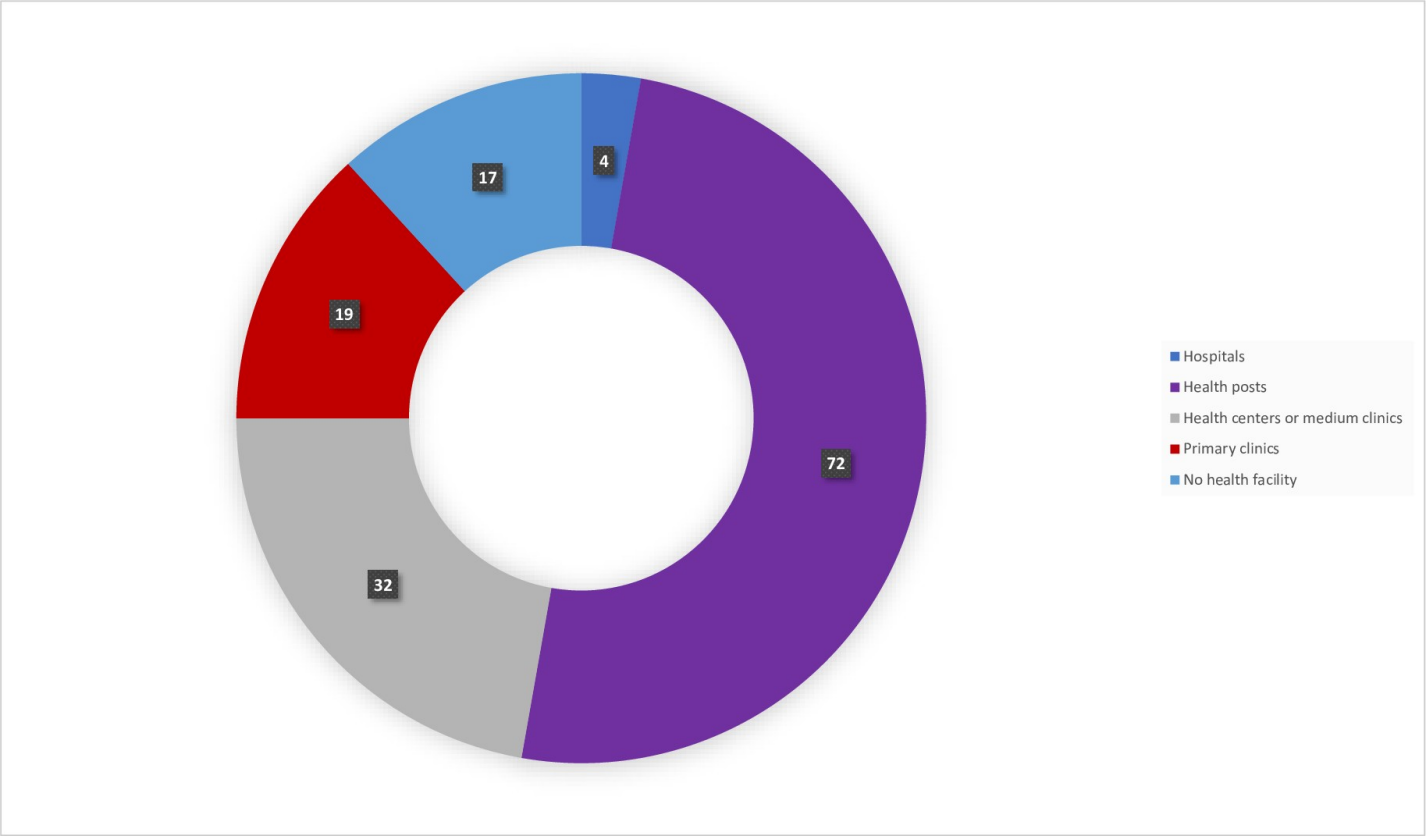
Type of health facilities accessed by enrolled organizations, October 2016 (N = 58).

This study revealed that out of 58 seasonal migrant worker employing organizations, over two-thirds had access to public health commodities essential for providing primary healthcare services including 4 (6.8%) hospitals, 17 (29.3%) health centers, 15 (25.8%) primary clinics, while the remaining 17 (30.0%) had no healthcare services within their compound, (Fig 2).

### Qualitative study findings

Access to malaria prevention and control interventions among seasonal migrant workers in seven of eleven regional states, 19 zones, 38 districts, and 58 workplaces was assessed, (Table 2). The qualitative data were collected from 7 seasonal migrant workers, 7 managers and health team leaders of seasonal migrant worker employing organizations, and 9 experts from health bureaus. The mean age of the interviewees was 37.7 (±SD 10.6) years. The study assessed large-scale government-owned farms and projects, large-scale private farms, small scale private farms, gold mining, and panning associations, and construction sites.

**Table 2:** Number of organizations, respondents, and key informants by region, zone and district, October 2016.

Ser. no.	Region	Zone	No. districts	No. employing organizations	No. respondents (M/F)*	No. key informants (M/F)
1	Afar	Zone 1	1	1	1/0	3/0
		Zone 3	2	2	2/0	
2	Amhara	Awi	2	2	2/0	2/0
		West Gojjam	1	1	1/0	
3	Benishangul Gumuz	Metekel Assossa	1	2	2/0	6/0
4	Gambella	Zone 1	1	2	2/0	2/0
		Zone 2	2	2	2/0	
5	Oromia	East Shoa	6	18	11/7	6/1
		Horo Gudru	1	1	1/0	
		Arsi	2	2	1/1	
		Jimma	1	1	1/0	
		Guji	1	1	1/0	
		Finifine Zuria	1	1	0/1	
		East Wollega	1	1	1/0	
6	SNNP	Dawuro	1	1	1/0	1/0
		Gamo Gofa	3	4	4/0	
		South Omo	4	11	10/1	
		Bench Mahi	1	2	2/0	
7	Tigray	Western	2	3	3/0	2/0
**Total**	**7**	**19**	**34**	**58**	**48/10**	**22/1**

NB: (M/F)*: Number of male and female key informants.

During the individual in-depth interviews, participants were asked to describe their access to malaria prevention and control interventions. The results and discussions of this qualitative data are presented under four themes and eight sub-themes (Table 3).

**Table 3:** Summary of themes and sub-themes.

Themes	Subthemes
Theme 1: Poor settlement facilities	-
Theme 2: Limited access and utilization of malaria prevention and control services	(a) Occupational risk of acquiring malaria
	(b) Limited access and utilization of LLINs
	(c) Lack of access to indoor residual spraying (IRS)
Theme 3: Limited access to curative health services	(a) Limitation in public health care services
	(b) Availability of a higher number of private health care services
	(c) Technical and financial support
Theme 4: Gaps and proposed solutions	(a) Challenges of health care services
	(b) Proposed interventions

#### Theme (1): Poor settlement facilities

**Fig 3:**
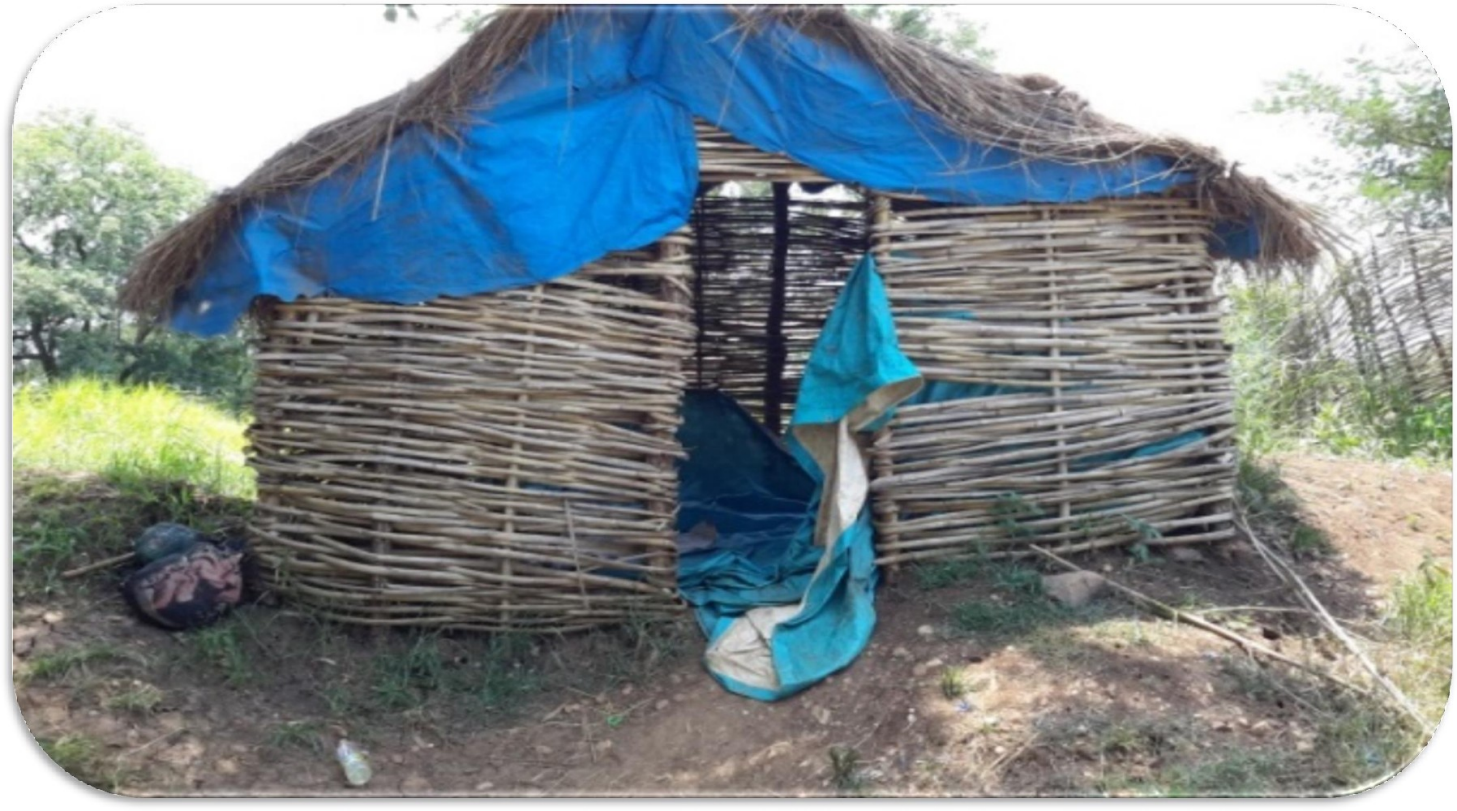
Temporary housing for seasonal migrant workers in Dangur, Benishangul Gumuz (photo: Argaw MD).

Housing standards including room size and construction materials varied depending on the type of employer. Some privately owned large-scale farms had settlement houses constructed primarily with bamboo structures and grass roofs while other large-scale government-owned or foreign investor-owned organizations had a permanent structure made of mud, cement, and agro-stones. The most common problem mobile and seasonal migrant workers faced in the destination areas include temporary housing made of bamboo and thatched roofs which presented difficulties in hanging LLINs in halls (Fig 3), and overcrowding. Large scale farms offered a 64 square meter room to accommodate an average of thirty seasonal migrant workers. The following verbatim illustrates the extent of overcrowding:

“On average, about 30 temporary workers should stay in a single room. Some of us leave the room and the space is difficult [from overcrowding] to properly hang mosquito bed nets.” [A seasonal migrant worker from a sugar plantation farm]

#### Theme (2): Limited access to malaria prevention and control services

*Subtheme (2a)*: *Occupational risk of acquiring malaria*. Employment categories such as sesame and sugar-cane plantations and watering farmlands demand that workers stay on the farm overnight. In addition, some seasonal migrant workers had longer working hours, serving for more than one employer. The following transcript illustrates this:

“…harvesting sesame seed is performed during the night since its capsule will open when it is exposed to the sunlight.… seasonal migrant workers spend eight hours working with us, and then during the night, they will work with another employer at private farms.” [A seasonal migrant worker supervisor from a sesame farm]

*Subtheme (2b)*: *Limited access to LLINs*. Half (29/58) of the assessed facilities had access to the free distribution of LLINs. In sugar cane plantations and factories, all permanent and seasonal migrant workers were targeted through community LLIN distributions. However, the private farms reported that they did not receive LLINs from government sources.

“…the district health office does not engage in LLIN distribution for our employees…they would tell us that they only target permanent residences.” [A manager from a seasonal migrant worker employing organization]

The limited utilization of available LLINs by the seasonal migrant workers was also among the main challenges. Seasonal workers did not bring back their LLINs with them during subsequent employments. In addition, there were misconceptions held by the workers regarding LLINs. Some believed LLINs prevent all pests other than mosquitos while others stated that LLINs are responsible for attracting bed bugs (*Cimex lectularius*) in sleeping rooms. The following transcript illustrates the issues around utilization:

“Sometimes we do not use chemically treated bed nets as it irritates the bed bugs.” [A seasonal migrant worker from sugar plantation farm]

*Subtheme (2c)*: *Limited access to indoor residual spray*. IRS is implemented by EFMOH in collaboration with its development partners (PMI, Global Fund, WHO, UNICEF), district health offices, community, and government projects. While the chemical and technical supports was from the district health office, the operational cost was covered by the EFMOH and development partners. However, there were found to be several factors that deterred the implementation of the IRS in seasonal migrant worker employment areas and housing such as: 1) IRS activities were not recommended in the area where organic sesame farms cultivated, 2) the structures of the housing (made of bamboo and grass) did not allow IRS, and, 3) temporary housings were not targeted for IRS interventions.

#### Theme (3): Limited access to curative health services

*Subtheme (3a)*: *Availability of limited number of public healthcare services*. All workplace hospitals and health centers diagnose and treat both uncomplicated and severe malaria cases, while health posts diagnose malaria using rapid diagnostic tests and treat only uncomplicated malaria cases. However, small-scale private-owned sorghum, sesame, vegetables, and horticulture farms (<500 hectares) did not have health facilities. This gap in health facilities was made worse by the fact that some farm owners or managers were engaged in selling anti-malarial drugs without testing, engaging in over-prescription of anti-malarial drugs for febrile illnesses, and would also deny sick leave and prompt medical care for seasonal migrant workers. The following quotes describe the situation:

“…*one of our patients reported that he took Coartem from his current employing organization*, *but the laboratory investigation reported relapsing fever*, *not malaria*…*” [A health team leader from a seasonal migrant worker employing organization]*

*Subtheme (3b)*: *Availability of a higher number of private healthcare services*. Private health facilities were available in the semi-urban areas of the destination districts of seasonal migrant workers. Some seasonal migrant workers sought malaria diagnosis and treatment services from private outlets. In addition, this study documented that some private farms lease medical services from nearby private health facilities.

“Within four zones of the Amhara region, where seasonal migrant workers have sought jobs, that is North Gondar, West Gojjam, Awi, and East Gojjam, there is one general hospital, 39 private medium clinics, and 344 private primary clinics.” [An expert from the regional health bureau]

*Subtheme (3c)*: *Technical and financial support*. Four out of seven regional state health bureaus strived to address the malaria prevention, control, and elimination related needs of seasonal migrant workers. Recruiting and deploying temporary health workers, assigning health extension workers, and establishing test and treat campaigns were some of the approaches used to address malaria in the seasonal migrant workers population. However, the test and treat campaign was interrupted due to lack of funds. One of the experts of the regional health bureau had the following to say:

“The passive malaria screening arranged by [name] zone health department through establishing temporary testing and treatment sites in bus stations were among the strategies executed to prevent the reintroduction of malaria in highland fringe districts. But, due to lack of budget, we couldn’t maintain provision of similar services elsewhere.” [An expert from a regional health bureau]

#### Theme (4): Gaps and proposed solutions

*Subtheme (4a)*: *Challenges of healthcare services*. Most seasonal migrant or mobile worker employing organizations that followed informal procedures exposed seasonal migrant workers to higher health risks through poor housing, nighttime work, and limited primary healthcare services. These organizations often do not disclose an accurate number of their employees which has become a challenge to implementing planned and effective interventions.

“Malaria is one of our district’s major public health problems. For example, the reports of the public health facilities in 2015/16 showed that there were over 32,557 cases with incidences of 508/1000 people per year. However, this incidence rate is a poor reflection of our actual challenges since the size of seasonal migrant workers is not well known. [An expert from a zone health department]

In addition, the small-scale projects and farms that employ seasonal migrant workers do not offer basic health services. Therefore, employees were forced to seek treatment from nearby healthcare facilities which they often unaffordable.

“We used to visit health posts and health centers to get a diagnosis for fever symptoms. Often, it was malaria or typhoid fever or something else. There are no anti-malarial drugs free of charge in both health posts and health centers and it is even much more expensive than private providers.” [A manager from a seasonal migrant worker-employing organization]

The malaria diagnosis and treatment and vector control interventions in some employing organizations did not follow national recommendations.

“In our camp, all malaria cases which consist of P. falciparum or P. vivax or clinical cases are treated with chloroquine.” [Aa health team leader from a seasonal migrant worker-employing organization]

Functional supply chain management systems ensure uninterrupted access to anti-malarial drugs and supplies. Unfortunately, the majority of seasonal migrant workers employing organizations reported that stockouts and frequently changing supply systems affect the quality of services in their facilities.

“We used to collect essential malaria drugs from health center and town health offices after submitting our filled drug report and request forms. We were informed that the drug supply system has been integrated into the Pharmaceutical Fund and Supply Agency (PFSA), so as we requested our drugs through them, we could not get any supplies as they were under inventory. Thus, we are facing increased malaria cases without any drugs at hand.” [A health team leader from a seasonal migrant worker-employing organization]

*Subtheme (4b)*: *Proposed intervention*. The participants indicated that the occurrence of malaria among seasonal migrant workers was much more complex and requires specific interventions at three levels: national, regional and large-scale farms or employers’ organization. The recommended interventions include: (1) leasing agreements with articles which address the tools and required services to be implemented at all levels with regards to health, safety and risk of migrant workers, (2) malaria programs that look for adequate resources to ensure availability of uninterrupted anti-malarial supplies, provision of malaria case management trainings, implementation of behavioral change communication, and deployment of health workers in these areas, and (3) the establishment of a system that forces seasonal migrant worker employing organizations to report the actual number of their employees.

## Discussion

Rapid growth and expansion of large-scale farms and construction sites attract the population into malaria-endemic areas [20]. In this formative assessment, we found that various development companies located in malaria endemic lowlands in the seven regions of Ethiopia attracted more than one million seasonal migrant mobile workers often from the less malaria endemic highlands. These seasonal migrant workers are at substantial risk to malaria infection.

Seasonal migrant workers lived in over-crowded and sub-optimal temporary housing facilities putting them at substantial risk of infectious diseases including malaria and other mosquito-borne conditions [21]. Housing structures with thatched and stick/mud roofs and earth/local dung plaster floors were previously significantly associated with positive malaria RDT results in Ethiopia [22]. Seasonal migrant workers lodge in these risky conditions for about 3–9 months and return to the historically low malaria transmission areas posing a complex challenge to the malaria prevention and control efforts in Ethiopia [4, 6].

Seasonal migrant workers in about half of the assessed facilities had limited access to malaria prevention and control interventions. Various factors might have contributed to this including lack of interventions targeting seasonal migrant workers, the high cost of LLINs in the free market, and seasonal migrants’ limited utilization of LLINs due to pests and difficulties in properly hanging the LLINs at sleeping places from the reported overcrowded living condition. A study in Myanmar, reported that the primary gap in malaria control among seasonal migrant workers was the lack of willingness on the part of seasonal migrant workers to buy LLINs due to affordability issues [23]. Poor knowledge on LLINs by seasonal migrant workers was another issue. Similarly, another study reported that out of 2,484 seasonal migrant workers, less than half had specific knowledge related to LLINs [21].

Public health facilities provide basic health services for permanent residents and seasonal migrant workers in the study areas with a limited number of health centers and health posts. Private for-profit facilities are the alternative service providers with a much larger presence in the community. However, the government planning for prevention and quantification of anti-malarial supplies only considers the permanent residents. In addition, some development organizations use an informal migrant worker employment system which made planning and intervention of services challenging. Thus, the large number of seasonal migrant and mobile workers employed in rural *kebeles* overwhelm the health facilities and cause frequent stock-out of essential drugs and supplies. This was consistent with the reports of a study in Ethiopia in which more than one-fourth of seasonal migrant workers suffered from typhoid fever, malaria, visceral leishmaniasis, HIV, and tuberculosis due to inadequate and sub-optimal medical services [24].

There were ongoing efforts to address malaria prevention, control, and elimination related needs of seasonal migrant and mobile workers. Some of the efforts include conducting test and treat campaigns and deployment of additional health workers for three to six months. This initiative was in line with the 61^st^ World Health Assembly resolution on the health of migrants (WHA 61.12), adopted in April 2008, which calls upon governments to “promote migrant-sensitive health policies” and to “promote equitable access to health promotion and care for seasonal migrants” [25]. However, the initiative was interrupted due to a lack of funds, coordination, and collaboration among multi-sectoral offices and development partners.

In this formative assessment, multi-sectoral collaboration has been cited as the main solution to address these complex challenges. National malaria prevention, control, and elimination activities in Ethiopia should address seasonal migrant worker commodity and service needs in their planning and budgeting to implement tailored malaria-related healthcare services for seasonal migrant worker populations. Large-scale farms should be responsible and required by law to fulfill basic healthcare, including malaria prevention and control interventions for their employees.

This formative assessment has limitations. First, the quantitative data on the number of migrant workers were collected from secondary employment records of the employer organizations, which may not have the records of all employees as some used informal employment structures. This may have underestimated the size of migrant workers. Second, the study focused on seven out of eleven administrative regional states of Ethiopia where the contexts vary. This may limit the generalizability of findings to the whole country. However, these seven regions are also the most malaria affected regions of the country and the data collected from these regions may still be able to provide an adequate picture of the situation.

## Conclusions and recommendations

Seasonal migrant workers in seven regions of Ethiopia are at substantial risk of acquiring malaria with poor access to malaria prevention, control, and elimination interventions. The movement of the workers between their worksites and home communities will lead to the resurgence of outbreaks of malaria in areas where transmission has been lowered. Coordinated actions led by a national multi-sectoral task force involving all stakeholders including Ministry of Agriculture, Ministry of Labor and Social Affairs and Ministry of Health is needed to a) map out seasonal migrant workers, their health service needs as well as stakeholders’ roles, and responsibilities, and b) develop appropriate and tailored malaria prevention control and elimination interventions in migrant seasonal worker destination districts.

## Supporting information

S1 File Data abstraction form. (XLSX)

S2 File Formative assessment tool on mobile and/or migrant workers and malaria. (DOCX)

S3 File Formative assessment tool on mobile and/or migrant workers and malaria. (DOCX)

S4 File Formative assessment tools for health, agriculture, investment, and labor and social affairs bureau. (DOCX)

S5 File (XLSX)

S6 File (XLSX)
