# Supplementary material for: Access to malaria prevention and control interventions among seasonal migrant workers: A multi-region formative assessment in Ethiopia
Source: PLoS One. 2021 Feb 23;16(2):e0246251. doi: 10.1371/journal.pone.0246251 (PMC7901780; doi:10.1371/journal.pone.0246251)
Supplement: S2 File — (DOCX) [file pone.0246251.s002.docx]

**S2 File. Formative assessment tool on mobile and/or migrant workers and malaria**

**Introduction**

This formative assessment tool is designed to explore the mobility dynamics and malaria related health care service available for migrant and/or mobile seasonal workers in seven regional states of Ethiopia. USIAD Private Health Sector Project will analyze the data and synthesis the information for evidence based decision making at various level. The result of this study will be used to identify and design malaria prevention and control interventions to address the health needs of migrant seasonal workers. Please note that the information you give us will be kept confidential and used only to fill the information gap at the health tire system.

### Formative Assessment: Key informant interview guides for regional health bureaus staff

Ice breaker

Greetings, introduction session & facilitating consent for participation

Step 1:- Main question

1. Please tell me the situation of malaria in the region and destination districts for migrant seasonal workers look for jobs?
2. How does the region/ woreda Health Office manage, prevent/control malaria in these areas? (Probe? Supply i.e. Arthimicinin Combined Therapy [ACT], Chloroquine, RDT, LLINs; set up temporary clinic etc.)
3. What are the major challenges you encountered in controlling and preventing malaria in work places? (Probe: ask, issue of malaria resurgence from returning migrant workers)
4. What are your plans to improve the situation of malaria in work place? What kind of solutions do you propose for each actors?

Step 2 :- Do you have any question for me?

Thank you so much!
