# Supplementary material for: Access to malaria prevention and control interventions among seasonal migrant workers: A multi-region formative assessment in Ethiopia
Source: PLoS One. 2021 Feb 23;16(2):e0246251. doi: 10.1371/journal.pone.0246251 (PMC7901780; doi:10.1371/journal.pone.0246251)
Supplement: S3 File — (DOCX) [file pone.0246251.s003.docx]

**S3 File. Formative assessment tool on mobile and/or migrant workers and malaria**

**Introduction**

This formative assessment tool is designed to explore the mobility dynamics and malaria related health care service available for migrant and/or mobile workers in seven regional states of Ethiopia. Private Health Sector Project will analyze the data and synthesis the information for evidence based decision making at various level of the health tire system. The result of this study will be used to identify and design malaria prevention and control interventions to address the health needs of risky group of population. Please note that the information you give us will be kept confidential and used only to fill the information gap at the health tire system.

### Formative assessment: health, agriculture, investment, and labor and social affairs bureau

**In-depth interview guides for seasonal migrant worker**

**Ice breaker**

1. Where did you come from? How long have you been living here? Did you move from your hometown alone or did you come with other family members? What business are you conducting here?
2. How many times do you go back to your hometown in a year? When was the last time you went back and when did you return? Why did you go back to your hometown? Do you go back regularly or was it due to illnesses, sickness or fever?
3. How has your health been since you moved here? How many times did you suffer from a fever in last three to six months? What remedies did you use? What are the most common diseases contracted amongst the group of people with whom you work or travel with? Are you aware of malaria? Do you know how malaria is transmitted? Do you know what can prevent malaria?
4. Have you ever suffered from malaria? How did you receive a testing, treatment or both for malaria? Who provided the testing, treatment or both and where was it conducted? How long did you suffer from malaria? Was the test, treatment or both free? Did you get treatment within 24 hours or later? Have you ever been tested before treatment? Do you know what kind of test was conducted and if so, what it was? Have you ever been admitted to hospital within this locality? How far did you need to travel to access the nearest health service delivery point or provider the last time you were sick? Were you able to afford all the healthcare costs (e.g. transportation, meals, tests, medicine) needed to cure your malaria? How much did it cost?
5. Do you know of personal protection materials that can prevent you from contracting malaria? What are they? Have you ever received or have personal protection materials been distributed during your period of residence here? Who supported you in accessing these materials? Was it free or did you have to pay for them?
6. Have you had any experiences that enable you to give suggestions to other workers on where they should go and what they should do when they are sick? How have you made decisions amongst yourselves whenever you are having health problems? If somebody amongst you is sick, what do other people or workers do and how do they help? 12. Have you heard of RDTs, ACTs, LLINs, ITNs, repellents, etc.? 13. Do you check your health status before you return to your hometown? Have you ever been tested for malaria before you returned to your hometown? Who tested you before you returned to your hometown or moved to another place other than your town of origin?

Thank you so much.
