# Supplementary material for: Access to malaria prevention and control interventions among seasonal migrant workers: A multi-region formative assessment in Ethiopia
Source: PLoS One. 2021 Feb 23;16(2):e0246251. doi: 10.1371/journal.pone.0246251 (PMC7901780; doi:10.1371/journal.pone.0246251)
Supplement: S4 File — (DOCX) [file pone.0246251.s004.docx]

## **S4 File. Formative assessment tools for health, agriculture, investment, and labor and social affairs bureau**

Formative assessment tool on mobile and/or migrant workers and malaria

**Introduction**

This formative assessment tool is designed to explore the mobility dynamics and malaria related health care service available for migrant and/or mobile workers in seven regional states of Ethiopia. Private Health Sector Project will analyze the data and synthesis the information for evidence based decision making at various level of the health tire system. The result of this study will be used to identify and design malaria prevention and control interventions to address the health needs of risky group of population. Please note that the information you give us will be kept confidential and used only to fill the information gap at the health tire system.

### Formative assessment: health, agriculture, investment, and labor and social affairs bureau

1. Inventory sheet of organizations and locations employing and deploying migrant/mobile workforce

Direction: The purpose of the table below is to have directory of organizations and their locations in the region so that the study team will go and do the rapid assessment on situation of malaria prevention and control.

Region:_______________________________________

Circle Source of the information ( Regional Health/Agriculture/investment Bureau:

| S. No. | Name of the organization employing and deploying migrant/mobile workers | Sector | Estimated size of workers | Location of the work place | |
| --- | --- | --- | --- | --- | --- |
|  |  |  |  | Zone/district | Kebele/village |
|  |  |  |  |  |  |
|  |  |  |  |  |  |
|  |  |  |  |  |  |
|  |  |  |  |  |  |
|  |  |  |  |  |  |
|  |  |  |  |  |  |
|  |  |  |  |  |  |
|  |  |  |  |  |  |
|  |  |  |  |  |  |
|  |  |  |  |  |  |

Key: Sector: Agriculture ( Sesame, cotton, sorghum, maize, others); Construction ( Dam, road, ); Mining ( gold, other), Other ( specify….)

1. PROFILE OF THE WORK PLACE

1.1 Interviewee’s description: Number Age:________ Sex:____

Position in the organization: Profession:____________________

Phone number:__________________________

1.2 Name of the organization

Office Address: Wereda HNo. Phone No.

1.3 Where is/are the work place located

Region Zone

Woreda Village:………………………………..

GIS coordinate

1.4 Please describe the sector your organization is currently actively working by employing migrant/mobile workers

A: Agriculture: 1. Sesame Farm 2. Sorghum Farm 3. Cotton Farm 4. Sugar cane plantation

B: Mining: 1 Gold mining and panning

C: Construction: 1. Dam, 2. Road C. factory D. other

7. Others: specify

1.5 Can tell us the estimated number and characteristics of works employed by your organization as described in the table below

| Month | Estimated number of employee | | | | Remark |
| --- | --- | --- | --- | --- | --- |
|  | Long term or permanent employee | | Temporary or mobile or migrant | |  |
|  | Male | Female | Male | female |  |
| July - September |  |  |  |  |  |
| October - December |  |  |  |  |  |
| January – March |  |  |  |  |  |
| April – June |  |  |  |  |  |

NB: Long term or permanent employee is worker who is working in the organization continuously for more than ……… months

Temporary or mobile/migrant workers who are employed seasonally and working for less than … months in the year

1.6 Where do majority of migrant workers come from? Pls tell us the region and district

Region: , district:

1.7 Where do the majority of the migrant/mobile workers go when upon termination of their

employment ?

1. return to their home villages/towns: yes No

2. stay in this village looking for other job : Yes No

3. I do not know

( Interviewer: document other remarks from the interviewee)

1.8 If you have permanent employees, where do the workers live?

A. Inside camp B. In the nearby town

1.9 If living inside a camp in the workplace, what is the typing of housing?

A. Temporary shelter

B. House (with permanent structure and roof)

C. Open field (outside)

D. Tent

E. Other (specify)

1.10. Where do majority of the migrant workers live during the work season ?

A. In side camp B. In the nearby town/village

1.11. Can you characterize the type of house in side camp ?

A.

B.

C.

1.12. How many workers do sleep together in one room?............ ( put range or average number)

1.13 When are the working hours of the migrant/mobile workers ?

i. Day time ii Night time iii. Both

1. On Access to Prevention and Control

2.1. Where do the workers access medical care when they are sick ?

A. The organizations clinic

B. nearby private facility

C. nearby public health facility

D. either B or C

2.2. If the organization has its own work place clinic, what is the level or type of health facility?

Circle which is appropriate: primary/medium/specialty clinic/specialty center/hospital

Health post/health center.

( Interviewer: if the organization has more than one facility, pls describe and characterize all types in terms of level and their distribution)

2.3. If your organization has a health facility, does it have the capacity?

A. to diagnose malaria with microscope or RDT or Both ( circle appropriate)

B. to treat uncomplicated malaria

C. to treat complicated and severe malaria

2.4. Where does your facility get antimalarial drugs and reagents from ?

A. organization procures from private distributers

B. gets regular supply of Anti-malaria drugs from Town Health Office [THO]/RHB/ Pharmaceutical Fund and Supply Agency [PFSA]

C. gets regular supply of staining reagents or RDT from THO/RHB/PFSA

2.5. Do your employees have Long Lasting Insecticide Treated Nets [LLIN] or Insecticide Treated Nets [ITN] ?

A; Yes B. No

2.6. If yes, where does your organization get the LLIN/ITN ?

A. the organization procures and distributes it to its employees

B. the organization is supplied by RHB/THO and distributes it to its employees

2.7. If answer to Q2.5 is no, why are they not utilizing

A. organization has financial limitation to buy and provide bed nets

B. RHB/THO has refused to provide bed net

C. other reason (specify) :-----------------------------------------

2.8. If your organization is providing bed nets to employees, how are you managing it ?

A. employees are given the bed net to be returned when they leave

B. employees are given the bed net and take it with them when they leave

C. the houses they live in have bed nets

d. Other: (Bed nets are sold at subsidized prices)

2.9. Tell us your challenge in managing bed nets?

2.10. Do the living quarters receive seasonal Indoor Residual Spray (IRS) services?

A. Yes, B. No.

2.11 If yes to Q 2.10, Who does provide the IRS ?

A. your organization

B. the town health offices

2.12 Do mobile/migrant workers receive routine testing and treatment for malaria before departing to

their home places ?

A. yes B. No

2.13. Tell us your organizations working relationship with THO or RHB in relation to malaria

prevention and control ?

- 1. Receive drugs A. yes B. No
  2. Receive reagents or RDT : A. yes B. No
  3. Receive bed nets: A. yes B. No
  4. Receive seasonal IRS: A. yes B. No
  5. Receive testing and treatment of cases when sick in public health facilities: A. yes B. No
  6. Submit performance or surveillance report: A. yes B. No

1. What kind of support does your organization need to address the malaria health needs of migrant workers?
   1. Case management :
   2. Malaria vector control

Thank you so much.
